# Filamentous prophage Pf4 promotes genetic exchange in *Pseudomonas aeruginosa*

**DOI:** 10.1093/ismejo/wrad025

**Published:** 2024-01-10

**Authors:** Tong-Tong Pei, Han Luo, Yuanyuan Wang, Hao Li, Xing-Yu Wang, Yi-Qiu Zhang, Ying An, Li-Li Wu, Junhua Ma, Xiaoye Liang, Aixin Yan, Liang Yang, Changbin Chen, Tao Dong

**Affiliations:** State Key Laboratory of Microbial Metabolism, Joint International Research Laboratory of Metabolic & Developmental Sciences, School of Life Sciences and Biotechnology, Shanghai Jiao Tong University, Shanghai 200240, China; Department of Immunology and Microbiology, School of Life Sciences, Southern University of Science and Technology, Shenzhen 518055, China; State Key Laboratory of Microbial Metabolism, Joint International Research Laboratory of Metabolic & Developmental Sciences, School of Life Sciences and Biotechnology, Shanghai Jiao Tong University, Shanghai 200240, China; Unit of Pathogenic Fungal Infection and Host Immunity, Key Laboratory of Molecular Virology and Immunology, Institute of Immunity and Infection, Chinese Academy of Sciences, Shanghai 200031, China; State Key Laboratory of Microbial Metabolism, Joint International Research Laboratory of Metabolic & Developmental Sciences, School of Life Sciences and Biotechnology, Shanghai Jiao Tong University, Shanghai 200240, China; Department of Immunology and Microbiology, School of Life Sciences, Southern University of Science and Technology, Shenzhen 518055, China; State Key Laboratory of Microbial Metabolism, Joint International Research Laboratory of Metabolic & Developmental Sciences, School of Life Sciences and Biotechnology, Shanghai Jiao Tong University, Shanghai 200240, China; Department of Immunology and Microbiology, School of Life Sciences, Southern University of Science and Technology, Shenzhen 518055, China; Department of Immunology and Microbiology, School of Life Sciences, Southern University of Science and Technology, Shenzhen 518055, China; Department of Immunology and Microbiology, School of Life Sciences, Southern University of Science and Technology, Shenzhen 518055, China; State Key Laboratory of Microbial Metabolism, Joint International Research Laboratory of Metabolic & Developmental Sciences, School of Life Sciences and Biotechnology, Shanghai Jiao Tong University, Shanghai 200240, China; State Key Laboratory of Microbial Metabolism, Joint International Research Laboratory of Metabolic & Developmental Sciences, School of Life Sciences and Biotechnology, Shanghai Jiao Tong University, Shanghai 200240, China; Department of Immunology and Microbiology, School of Life Sciences, Southern University of Science and Technology, Shenzhen 518055, China; School of Biological Sciences, The University of Hong Kong, Hong Kong Special Administrative Region 999077, China; School of Medicine, Southern University of Science and Technology, Shenzhen 518055, China; Unit of Pathogenic Fungal Infection and Host Immunity, Key Laboratory of Molecular Virology and Immunology, Institute of Immunity and Infection, Chinese Academy of Sciences, Shanghai 200031, China; Nanjing Advanced Academy of Life and Health, Nanjing 211135, China; Department of Immunology and Microbiology, School of Life Sciences, Southern University of Science and Technology, Shenzhen 518055, China

**Keywords:** protein secretion, prophage, interspecies interaction, biofilm

## Abstract

Filamentous prophages are widespread among bacteria and play crucial functions in virulence, antibiotic resistance, and biofilm structures. The filamentous Pf4 particles, extruded by an important pathogen *Pseudomonas aeruginosa*, can protect producing cells from adverse conditions. Contrary to the conventional belief that the Pf4-encoding cells resist reinfection, we herein report that the Pf4 prophage is reciprocally and commonly exchanged within *P. aeruginosa* colonies, which can repair defective Pf4 within the community. By labeling the Pf4 locus with antibiotic resistance and fluorescence markers, we demonstrate that the Pf4 locus is frequently exchanged within colony biofilms, in artificial sputum media, and in infected mouse lungs. We further show that Pf4 trafficking is a rapid process and capable of rescuing Pf4-defective mutants. The Pf4 phage is highly adaptable and can package additional DNA doubling its genome size. We also report that two clinical *P. aeruginosa* isolates are susceptible to the Pf4-mediated exchange, and the Pf5 prophage can be exchanged between cells as well. These findings suggest that the genetic exchanging interactions by filamentous prophages may facilitate defect rescue and the sharing of prophage-dependent benefits and costs within the *P. aeruginosa* community.

## Teaser

Microbial communities are crucial ecological entities with complex interactions, among which metabolites and genetic exchange can dictate community composition and stability. Previous studies have focused on metabolites excreted by one cell and used by another. Here, we demonstrate that the Pf4 prophage is frequently exchanged among *P. aeruginosa* cells, enabling the effective repair of Pf4-defective cells. Given that *P. aeruginosa* cells build better biofilms and gain increased virulence and antibiotic resistance at the cost of producing phage particles, Pf4 trafficking might effectively prevent potential cheating behaviors and ensure cost-sharing within the population.

## Introduction

Microbes form intricate communities in natural and host environments, playing pivotal roles in diverse functions including microbiota homeostasis for human health, environmental remediation, and pathogen–host interactions [1–4]. Their sophisticated interactions, encompassing both competition and cooperation, dictate microbial community structure and dynamic changes [5–8]. Although competition is crucial for community composition [9], cooperation has also emerged as a key driver of community stability and evolution [10, 11]. In addition, microbial communities host an even greater number of bacteriophages, adding another dimension of interactions among microbes [12]. However, phage-mediated interactions are generally considered an infectious process rather than a recurring form of cell–cell interactions.

*Pseudomonas aeruginosa* is an important pathogen that can cause serious wound and lung infections, especially in immune-compromised and cystic fibrosis patients [13–16]. In addition to synthesizing a plethora of virulence factors, including small molecules and secreted toxins, *P. aeruginosa* also adopts a robust biofilm lifestyle to survive antibiotic treatment and host immune response during infection [13, 17–21]. The *P. aeruginosa* biofilm contains not only common components including extracellular polysaccharides, proteins, and DNA, but also filamentous crystalline-like particles produced by the Pf endogenous prophages [22–24]. A Pf filament comprises an outer protein coating and an enclosed single-strand DNA [25, 26], but some DNA-free Pf filaments have recently been found [26]. Many clinical strains of *P. aeruginosa* also encode the Pf prophage genes [27–31].

The Pf4 prophage is encoded by a 12-kb gene cluster in the *P. aeruginosa* type strain PAO1 [32]. Activation of the Pf4 prophage is collectively controlled by host-encoded proteins, including two H-NS-family repressors, MvaT and MvaU [33], regulators for DNA repair and oxidative stress [34, 35], a two-component regulator, BfmR [36], as well as Pf4-encoded proteins, including a repressor, Pf4r [37, 38], an excisionase, XisF4 [37], and a toxin-antitoxin pair, PfiT-PfiA [39]. Importantly, this multifaceted regulation ensures that Pf4 remains inactive until cells sense environmental cues. Indeed, Pf genes are among the highest induced genes in *P. aeruginosa* biofilms and during anaerobic growth [25, 40–42]. Although Pf phages can be continually extruded by cells, the extruded phages normally do not kill producing cells or lyse the biofilms [25, 40, 41, 43]. The Pf4 filaments not only contribute to biofilms as structural components but also act as an iron chelator and inhibitor of fungal growth, a physical barrier against antibiotics, and an important immune modulator during infection [21, 22, 26, 44–46].

The Pf4 uses the type IV pili of *P. aeruginosa* as a surface receptor for infection [47]. Pulled into the periplasm by pili retraction, the phage filament interacts with an inner-membrane receptor, TolA, and releases its ssDNA into the cytoplasm while the filament coating proteins are retained in the inner membrane [48]. The ssDNA is then replicated into a circular double-strand DNA, which can be either integrated into the genome or maintained as a plasmid [21, 41]. In addition, the lysogenized Pf4 prophage is believed to confer *P. aeruginosa* PAO1 resistance to infections by Pf4 phages because only the Pf4-deletion mutant but not the wild type can be lysed by Pf4 [25]. The Pf4-capsid proteins could prevent superinfection by interfering with the function of type IV pilus [49, 50]. However, superinfections may occur and result in cell lysis when the Pf4 genome is mutated or the number of Pf4 phages overwhelms the host tolerance, but the mechanism of superinfection is not fully understood [25, 34, 38, 47].

Here, we demonstrate that the Pf4 locus is frequently exchanged among *P. aeruginosa* cells under *in vitro* biofilm-forming conditions and mouse-lung infection conditions. By labeling Pf4 with antibiotic-resistance, fluorescence, or luminescence reporters, we report that Pf4 could be transferred among *P. aeruginosa* cells at the fraction of about 0.01% on lysogeny broth (LB) colony biofilms, and the transfer could be stimulated to 1% under Pf4-activating conditions. A Pf4 structural-gene-deletion mutant could be rescued by Pf4-functional neighboring cells. Pf4 is capable of packaging additional DNA to double its genome size, and the transferred Pf4 could exist as circular DNA or in the form of chromosomal integration. We also detected similar exchanges in clinical *P. aeruginosa* isolates and in the Pf5-prophage strain. These findings highlight that the filamentous phage may serve as a common good undergoing frequent exchanges among *P. aeruginosa* cells, resembling a specialized form of intraspecies interactions that ensures Pf4-mediated protective functions are preserved and contributed by community members.

## Materials and methods

### Bacterial strains and growth conditions

Strains, plasmids, and primers used in this study are listed in Supplementary Tables S1–S3, respectively. Strains were grown in LB ([w/v] 1% tryptone, 0.5% yeast extract, 0.5% NaCl) aerobically at 37°C unless otherwise stated. The ASM was prepared as previously described [51]. Antibiotics were used at the following concentrations: streptomycin (100 μg/ml), irgasan (25 μg/ml), carbenicillin (100 μg/ml), gentamicin (20 μg/ml), tetracycline (75 μg/ml).

### Pf4 and Pf5 transfer assays

Donor and recipient cells were collected from overnight LB-agar plates and resuspended in fresh liquid LB. Cells were harvested by centrifugation at 4500 × *g* for 3 min, resuspended in fresh LB to OD_600_ ~ 1 and then mixed at a ratio of 1:1 (donor:recipient). For the transfer assay using liquid media, PAO1 mixtures were then subcultured into fresh liquid LB or ASM at a ratio of 1:100 and grown at 37°C for 12 h. For the transfer assay using solid media, the cell mixtures were spotted on LB-agar plates for 24 h at 37°C unless otherwise stated. For the contact-independent transfer assay, recipient cells were initially spotted on LB-agar plates. After air-drying the bacterial solution, a 0.22 μm nitrocellulose membrane was placed over it, and the donor cells were spotted on the same location as the recipients. The plates were then incubated for 24 h at 37°C. Cells were then resuspended in 500 μl LB, and a series of 10-fold dilutions were plated on LB plates with appropriate antibiotics.

### Fluorescence microscopy

Cells were collected from overnight LB-agar plates and resuspended in fresh liquid LB. The cells were then concentrated to OD_600_ ~ 10 and mixed as indicated or not. The cultures were then spotted on the 1% agarose pad in 0.5 × phosphate buffer saline (PBS) buffer, followed by fluorescence microscopy. Imaging was performed using the Nikon Ti2-E inverted microscope, equipped with the Perfect Focus System and a Plan Apo 100x Oil Ph3 DM (NA 1.45) objective lens. Fluorescence excitation utilized ET-GFP (Chroma #49002) and ET-mCherry (Chroma #49008) filter sets. Fiji software (2.3.0) was used to process images.

### Twitching motility assay

The assay was performed as previously described with minor modifications [52]. PAO1 strains were initially cultured by streaking onto 1.5% LB-agar plates and incubated overnight at 37°C. To assess twitching motility, bacterial cells were then inoculated by stabbing through a 1% LB-agar plate down to the underlying plastic dish. Following another 48-h incubation at 37°C, the agar was gently removed, and the extent of motility on the plastic surface was visualized after staining with Coomassie brilliant blue.

### Animal experiment

Cells were collected from overnight LB-agar plates and resuspended in 0.5 × PBS buffer. The bacterial cells were then washed with the 0.5 × PBS buffer and adjusted to 1 × 10^9^ c.f.u./ml. The donor and recipient cells were mixed together at a ratio of 1:1. Each 6–8-weeks-old female BALB/c mouse was anesthetized after treatment with 7.5% chloral hydrate. Then 20 μl bacterial suspension was intranasally inoculated into each mouse. After a 16-h infection, the mice were sacrificed by CO_2_, and the lungs were isolated, followed by homogenization using 1% proteose peptone. The bacterial loads were determined by a series of 10-fold dilutions on LB plates with appropriate antibiotics.

### Software

Prism 9.3.0 was used for all statistical analyses. Source data are provided as a Source Data file. The model figure was generated using BioRender (https://biorender.com).

## Results

### Pf4 locus is transferred among *P. aeruginosa* cells

**Figure 1:**
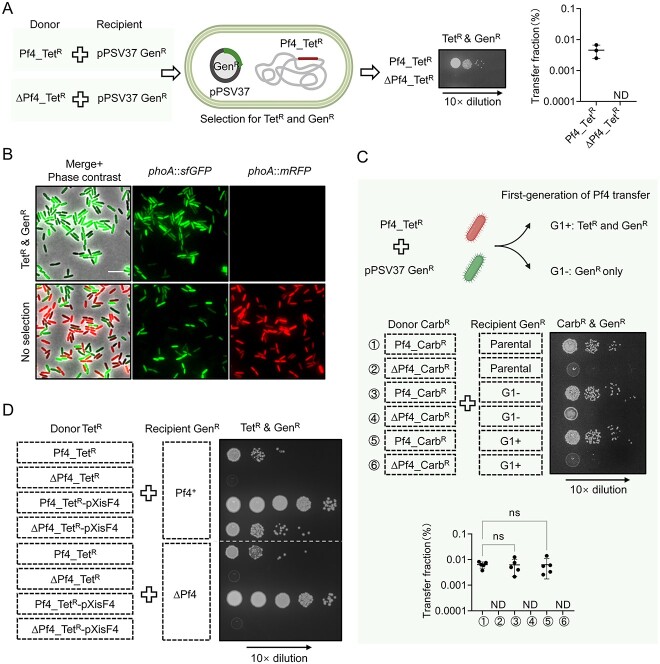
Characterization of the Pf4 transfer; (A) Pf4 transfer was observed among PAO1 cells; PAO1 cells labeled with tetracycline (Tet^R^) and gentamicin (Gen^R^) resistance carry Tet^R^ genes in the Pf4 locus and the pPSV37 plasmid, respectively; the same labeling applies to the rest of the figures unless otherwise specified; the transfer fraction was calculated as the ratio of daughter cells with dual resistance to tetracycline and gentamicin to all cells with gentamicin resistance in each sample; error bars indicate the standard deviation of three biological replicates; ND, not detected; (B) fluorescence microscopy of isolated PAO1 daughter cells; the parental PAO1 strains included *phoA*::*mRFP* Pf4_Tet^R^ as the donor and *phoA*::*sfGFP* carrying pPSV37 plasmid as the recipient; the daughter cells were collected from the overnight LB-agar plates with or without antibiotic selection as indicated, and incubated in LB with 1 mM IPTG for 30 min prior to imaging; cells were placed on agarose pads (~0.13 mm thick) for imaging to restrict their movement; a 30 × 30 μm representative field of cells is shown; scale bar, 5 μm; (C) Pf4 transfer among PAO1 daughter cells; top: schematic showing isolated G1+ and G1− daughter cells; middle: Pf4 transfer assay between PAO1 Pf4_Carb^R^ strains with initial PAO1 Gen^R^, G1+ or G1− daughter cells, respectively; bottom: quantification of the Pf4 transfer; the transfer fraction was calculated as the ratio of daughter cells with dual resistance to carbenicillin and gentamicin to all cells with gentamicin resistance in each sample; error bars indicate the standard deviation of five biological replicates, and statistical significance was calculated using one-way ANOVA (analysis of variance) test; ns, not significant; ND, not detected; (D) Pf4 transfer assay between different PAO1 mutants; the plasmid-borne excisionase XisF4 (pXisF4) was used to stimulate Pf4 production; for (A), (C), and (D), PAO1 ∆*PA0717–PA0726* mutants were used as the ∆Pf4 control.

To detect Pf4 transfer among *P. aeruginosa* cells, we constructed Pf4-labeled donor cells (Pf4_Tet^R^) by inserting a tetracycline-resistance (Tet^R^) cassette between the *pfiT* gene and the *attR* site, which is located at the end of the Pf4 gene cluster to avoid any pleiotropic effects on the native transcription of the Pf4 cluster (Fig. 1A, Supplementary Fig. 1A). To differentiate donor and recipient cells, we transformed recipient PAO1 cells with the pPSV37 plasmid that confers gentamicin resistance (Gen^R^) (Fig. 1A). As a negative control, we also constructed a defective ∆Pf4_Tet^R^ mutant by deleting genes *PA0717*–*PA0726*, which lacks most of the Pf4 essential components, including the ssDNA binding protein, capsid proteins, and the zot protein. The Pf4-transfer-positive daughter cells were expected to be resistant to both tetracycline and gentamicin. Through antibiotic selection, we found that double-resistant mutants were readily obtained, at a fraction of about 0.01% of recipient cells, while no such mutants were obtained in the ∆Pf4_Tet^R^ sample (Fig. 1A, Supplementary Fig. 1B).

To confirm that the gain of double resistance was due to transferred Pf4 but not the pPSV37 plasmid, we employed fluorescence microscopy analysis. To differentiate donor and recipient cells, we constructed a Pf4_Tet^R^*phoA*::*mRFP* mutant as the donor, in which the *P_lac_* promoter and *mRFP* gene were chromosomally inserted replacing the alkaline phosphatase gene *phoA*, and used the PAO1 pPSV37 *phoA*::*sfGFP* strain as the recipient, by replacing the *phoA* gene with the *P_lac_* promoter and *sfGFP* gene. After the transfer assay, all Tet^R^ and Gen^R^ double-resistant mutants exhibit green fluorescent protein (GFP)-only signals (Fig. 1B, Supplementary Fig. 1C). Collectively, these data indicate that Pf4 can be transferred among PAO1 cells within colonies during routine growth on LB plates.

### Pf4 is repetitively transferred among PAO1 cells

Because only a portion of recipient cells became Pf4_Tet^R^ after the transfer assay, we next tested whether these Pf4-acquired recipient cells are more prone to transfer, while the nontransferred cells are naturally resistant to Pf4. Using the Pf4_Tet^R^ as the donor in a transfer assay, we isolated daughter cells with or without Tet^R^, the former named first-generation G1+ cells and the latter G1− cells, which were used for a subsequent round of transfer assay (Fig. 1C) with a Pf4_Carb^R^ (carbenicillin resistance) donor. We constructed the Pf4_Carb^R^ donor by replacing the *tetR* and *tetA* genes with the *P_ampR_* promoter and *ampR* gene in the Pf4 gene cluster. After the co-incubation of donor and recipient pairs, G1+ and G1− recipient cells exhibited the same efficiency for acquiring Carb^R^ as their parental cells (Fig. 1C, Supplementary Fig. 1D). These results suggest that the observed Pf4 transfer is intrinsic but not due to genetic mutations of a subpopulation.

### Rescue of defective Pf4 through reinfection with wild-type phage

To test the efficiency of Pf4 transfer under a Pf4-inducible condition, we next employed the expression of XisF4, a Pf4-encoded excisionase that could promote Pf4 prophage excision and particle production [37]. Although *xisF4* is cloned downstream of an arabinose-inducible promoter, we found that basal expression of XisF4 without induction increased the fractions of Pf4 transfer by about 100-fold relative to the no-plasmid wild-type control (Fig. 1D, Supplementary Fig. 1E). Unexpectedly, the ∆Pf4 donor expressing XisF4 produced a number of Tet^R^ and Gen^R^ double-resistant daughter cells comparable to the parental donor, while we obtained no double-resistant daughter cells when both donor and recipient cells were Pf4-defective (Fig. 1D, Supplementary Fig. 1E). These results not only indicate that Pf4 transfer can be stimulated under Pf4-activating conditions but also suggest that the ∆Pf4 donor can be rescued by the transferred wild-type Pf4 from sister cells.

### Transferred Pf4 could be detected in multiple flexible forms

**Figure 2:**
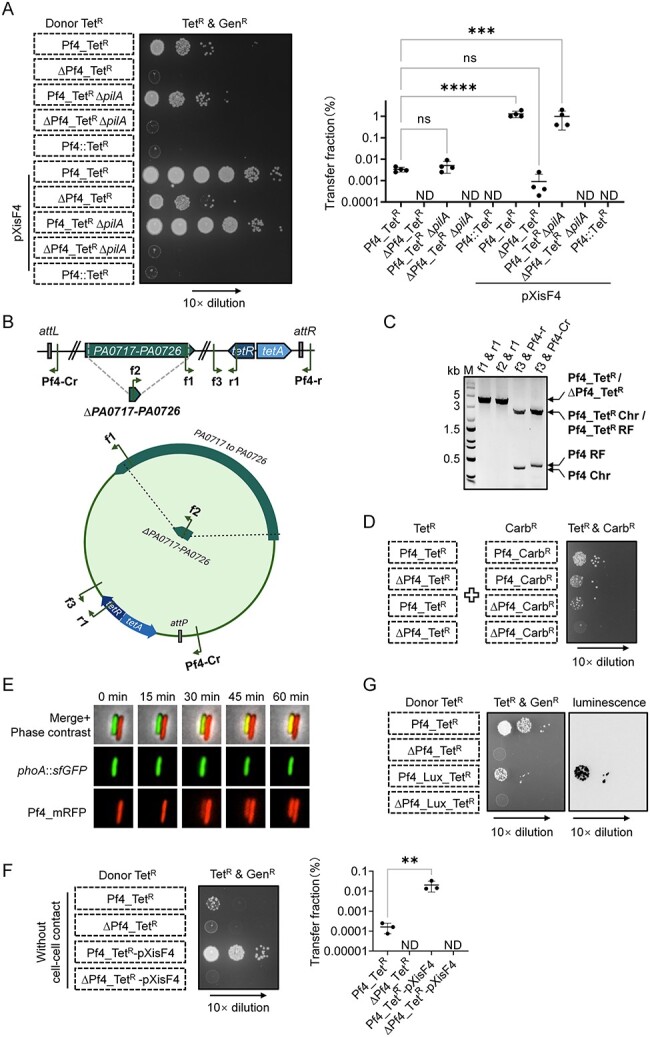
The transfer of Pf4-defective variants could be rescued by functional Pf4; (A) Pf4 transfer assay evaluating the Pf4 transfer efficiencies in different PAO1 mutants; the pXisF4 was used to stimulate Pf4 production; in the Pf4::Tet^R^ cells, the whole Pf4 cluster was replaced with a tetracycline-resistance cassette in the chromosome; the transfer fraction was calculated as the ratio of daughter cells with dual resistance to tetracycline and gentamicin to all cells with gentamicin resistance in each sample; error bars indicate the standard deviation of four biological replicates, and statistical significance was calculated using one-way ANOVA test, ^***^P < .001, ^****^P < .0001; ns, not significant; ND, not detected; (B) schematic showing the Pf4 prophage in the PAO1 chromosome and the Pf4 RF in the cytoplasm of PAO1; the PCR primers used in (C) are shown as arrows; (C) PCR amplification of Gen^R^ and Tet^R^ double-resistant daughter cells from PAO1 carrying pPSV37 and ∆Pf4_Tet^R^ carrying pXisF4 with primers as indicated; Chr, chromosomally integrated; (D) Pf4 transfer assay between PAO1 Pf4_Tet^R^ and Pf4_Carb^R^ mutants; (E) fluorescence microscopy of PAO1 Pf4_mRFP carrying pXisF4 mixed with *phoA*::*sfGFP* cells for 1 h; cells were placed on agarose pads (~0.13 mm thick) for imaging to restrict their movement; a 5 × 5 μm representative field of cells is shown; (F) Pf4 transfer assay evaluating the cell–cell-contact-independent Pf4 transfer among PAO1 cells; error bars indicate the standard deviation of three biological replicates, and statistical significance was calculated using one-way ANOVA test, ^**^P < .01; ND, not detected; (G) Pf4 transfer assay evaluating the transfer efficiencies of Pf4 variants with expanded genomes among PAO1 cells; for (A), (F), and (G), the donor strains are indicated, while the recipient is PAO1 carrying pPSV37; for (A), (C), (D), (F), and (G), PAO1 ∆*PA0717–PA0726* mutants were used as ∆Pf4 controls.

The rescuing phenotype by sister cells is surprising since most Pf4 genes were deleted in the ∆Pf4 mutant. To determine how the transferred Pf4 from sister cells could support the transfer of Tet^R^ in the ∆Pf4 donor, we first tested the role of PilA, a known pili receptor of Pf4. Using a panel of donor strains and the wild-type PAO1 as the recipient, we found that the deletion of *pilA* abolished the rescuing phenotype in the ∆Pf4_Tet^R^ samples (Fig. 2A, Supplementary Fig. 2A). We also found that the PilA pili were not required for Pf4 extrusion, as Pf4_Tet^R^ ∆*pilA* donor cells produced double-resistant cells at parental levels (Fig. 2A). In addition, when we replaced the whole Pf4 cluster with a tetracycline-resistance cassette in the donor Pf4::Tet^R^, no double-resistance daughter cells were obtained (Fig. 2A). This result suggests that the expression of XisF4 selectively promotes the transfer of Pf4 without affecting the transfer of other chromosomal genes.

**Figure 3:**
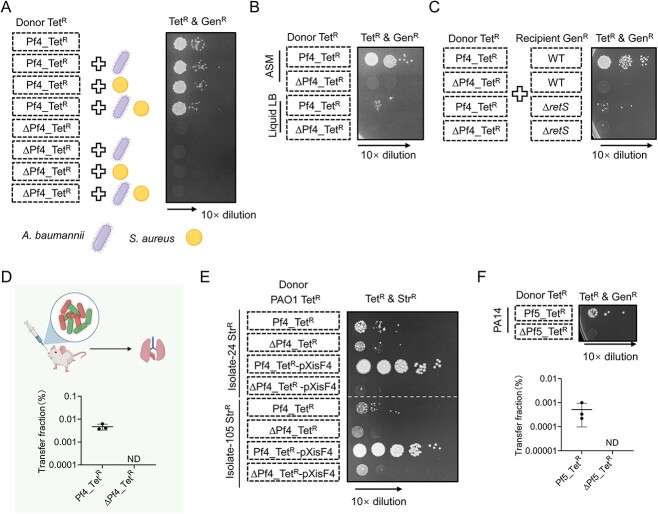
Pf4 transfer occurs under multiple conditions; (A) Pf4 transfer assay between different PAO1 populations in a multispecies environment; the donor strains of PAO1 mixtures are indicated, while the recipients were PAO1 strains carrying pPSV37; PAO1 mixtures were co-incubated with or without other species as specified; (B) Pf4 transfer assay between different PAO1 populations using liquid LB medium or ASM as indicated; (C) Pf4 transfer assay using PAO1 wild type (WT) strains or ∆*retS* mutants as recipients; (D) Pf4 transfer assay between different PAO1 populations using the mouse lung infection model; (E) Pf4 transfer assay between PAO1 Pf4_Tet^R^ mutants and *P. aeruginosa* clinical isolates with streptomycin-resistant (Str^R^); (F) Pf5 transfer assay between PA14 strains; PA14 cells were labeled with Tet^R^ inserted in the Pf5 locus and Gen^R^ conferred by the pPSV37 plasmid, respectively; ∆Pf5, the PA14 ∆*EIP97_20555-EIP97_20600* mutant; for (D) and (F), the transfer fraction was calculated as the ratio of daughter cells with dual resistance to tetracycline and gentamicin to all cells with gentamicin resistance in each sample; Error bars indicate the standard deviation of three biological replicates; ND, not detected; For (A) to (E), PAO1 ∆*PA0717–PA0726* mutants were used as ∆Pf4 controls.

We further investigated how the rescuing phenotype could occur. We postulated that, after the wild-type Pf4 is transferred to the ∆Pf4 mutant, the defective ∆Pf4 could be packaged by wild-type-encoded components and transferred back to the donor strain, followed by either chromosomal integration or existing in the circular replicative form (RF). To detect these Pf4 variants, we designed four primer pairs targeting internal Tet^R^ and ∆Pf4 regions, the integration *attR* site, as well as the end-joined region unique to the circular RF (Fig. 2B). We first confirmed that these primer pairs could amplify the target sequences and detect both chromosomally integrated Pf4 and circular Pf4 (Supplementary Fig. 2B–F). Then, we used these primer pairs to test the Gen^R^ and Tet^R^ double-resistant daughter cells resulting from the co-incubation of PAO1 cells carrying the pPSV37 plasmid (Gen^R^) and ∆Pf4_Tet^R^ carrying the plasmid-borne excisionase XisF4 (pXisF4) plasmid (Fig. 2C). To capture all possible Pf4-existing forms, we used a mixture of these daughter cells as the PCR template. We detected polymerase chain reaction (PCR) signals corresponding to not only parental chromosomal and circular Pf4 but also parental ∆Pf4_Tet^R^, recombined Pf4_Tet^R^, and chromosomally integrated and circular Tet^R^ regions (Fig. 2C). These results suggest that the transferred Pf4 may undergo recombination or exist independently in the variant form of circular DNA or chromosomal integration.

To determine whether Pf4 variants could stably co-exist in the same cell, we mixed Pf4_Tet^R^ and Pf4_Carb^R^, as well as the corresponding ∆Pf4 mutants, in different pairs. Results show that Tet^R^ and Carb^R^ double-resistant daughter cells could be readily isolated, in contrast to the ∆Pf4-negative control (Fig. 2D, Supplementary Fig. 2G). These acquired mutants could maintain Pf4 Tet^R^ and Carb^R^ variants for at least 24 h without antibiotic selection (Supplementary Fig. 2H). Collectively, these results highlight the flexibility of Pf4 trafficking among cells.

### Pf4 trafficking is rapid and amenable to package cargo DNA

We next tested how fast Pf4 transfer could occur among cells. To detect Pf4 transfer, we employed the Pf4_mRFP strain carrying pXisF4 as the donor. In this donor strain, we inserted the *mRFP* gene into the Pf4 cluster between the *pfiT* gene and the *attR* site. The *phoA*::*sfGFP* strain served as the recipient for imaging the transfer of Pf4_mRFP into sfGFP-positive recipients. After mixing the cells on an agarose pad for 30 min, we detected mRFP signals in recipient cells, both in the presence and absence of direct contact with donor cells (Fig. 2E, Supplementary Fig. 3A). These data indicate that the transfer event is a rapid process and could occur in a contact-independent manner. To confirm the occurrence of Pf4 transfer in the absence of direct cell–cell contact, we employed a 0.22 μm nitrocellulose membrane to physically separate donor and recipient strains in a co-incubation assay. We observed that the Pf4_Tet^R^ donor strains exhibited a transfer fraction of ~0.0001%, while the Pf4_Tet^R^ donor strains carrying pXisF4 exhibited a transfer fraction of about 0.01% (Fig. 2F, Supplementary Fig. 3B). Although there is about a 100-fold reduction relative to the no-membrane-separation conditions, these results indicate that the Pf4 transfer does not require cell–cell contact.

To test whether the Pf4 cluster is amenable to package additional DNA, we inserted an 8.5-kb fragment containing the *luxCDABE* operon under the control of a constitutive *rplK* promoter upstream of the *tetR* gene within the Pf4 cluster (Supplementary Fig. 3C). Note that the wild-type genome of Pf4 is around 12 kb, while the added size of the *luxCDABE* cluster and the *tetR* and *tetA* genes is about 8.5 kb. Transfer assays show that the Pf4_Lux_Tet^R^ could also be transferred, albeit at a reduced rate of about 10-fold less than the Pf4_Tet^R^. These results indicate that the Pf4 phage can accommodate a substantial genome size increase (Fig. 2G, Supplementary Fig. 3C).

### Pf4 trafficking can occur in polymicrobial and infection conditions

Because *P. aeruginosa* is an important pathogen that can cause skin and lung infections, we next asked whether Pf4 transfer could occur in clinically relevant conditions. First, we tested the effect of other bacteria on Pf4 transfer by mixing *P. aeruginosa*, *Acinetobacter baumannii*, and *Staphylococcus aureus* on LB plates in pairs or all together in a group. These pathogens are commonly associated with biofilm-associated wound infections similar to *P. aeruginosa* [53]. We found that the fraction of Pf4 transfer among PAO1 cells was not affected by the presence of these two pathogens (Fig. 3A, Supplementary Fig. 4A).

Since *P. aeruginosa* may cause chronic lung infections in cystic fibrosis patients, we next tested whether Pf4 transfer could occur in the artificial sputum medium (ASM), which is a viscous liquid environment promoting biofilm formation [51]. Results show that Pf4 transfer was 100-fold more efficient in ASM than in liquid LB culture, suggesting that Pf4 transfer may be stimulated *in vivo* (Fig. 3B, Supplementary Fig. 4B). To determine how the Pf4 transfer is regulated, we tested the effect of RetS, a sensor kinase and master regulator of virulence and type IV pili in *P. aeruginosa* [54]. Recipient ∆*retS* mutants exhibited severely impaired Pf4 transfer, suggesting that RetS is critical for Pf4 trafficking (Fig. 3C, Supplementary Fig. 4C). We performed a twitching motility assay and confirmed that the ∆*retS* mutant displayed impaired twitching, similar to the ∆*pilA* mutant (Supplementary Fig. 4D).

We further tested whether Pf4 transfer could occur during infection in mice using a lung infection model [55]. Mice were subjected to infection with PAO1 pPSV37 recipient strains together with either PAO1 Pf4_Tet^R^ or the ∆Pf4_Tet^R^ donor strains. We observed Pf4 transfer in samples containing Pf4_Tet^R^ strains at a comparable level to *in vitro* conditions, but not in the ∆Pf4_Tet^R^ mutant group (Fig. 3D). Collectively, these results indicate that Pf4 transfer can occur in polymicrobial and infection conditions.

### The trafficking of Pf4 and Pf4-like prophages occurs in clinical isolates

To determine whether the exchange of Pf4 is restricted to PAO1, we selected several *P. aeruginosa* clinical isolates as recipients and tested whether they could acquire Pf4. These isolates were collected from the sputum samples of ventilator-associated pneumonia patients [56]. To differentiate donor and recipient cells, we isolated the streptomycin-resistant (Str^R^) mutants of clinical isolates and used them as recipients. We obtained clinical strains No. 24 and No. 105 with Pf4_Tet^R^ after co-incubation with PAO1 Pf4_Tet^R^ donors (Fig. 3E, Supplementary Fig. 4E). The basal expression of plasmid-borne XisF4 stimulated the Pf4 transfer by 100-fold (Fig. 3E). The *P. aeruginosa* strain PA14 contains a filamentous prophage Pf5 integrated into its chromosome [57]. To detect Pf5 transfer, we constructed a donor strain by introducing a tetracycline-resistance cassette into the end of the Pf5 gene cluster (Pf5_Tet^R^) and transformed recipient PA14 cells with the pPSV37 plasmid that confers Gen^R^. As a control, we generated a nonfunctional ∆Pf5_Tet^R^ mutant by deleting genes *EIP97_20555*-*EIP97_20600* that encode Pf5 essential components. The results showed that Pf5_Tet^R^ could also be transferred between PA14 strains (Fig. 3F, Supplementary Fig. 4F).

## Discussion

Understanding the lifestyle of *P. aeruginosa* is a critical step toward developing effective treatment strategies for controlling its infection. Prior to this study, wild-type PAO1 is shown to be resistant to Pf4 reinfection [25, 38], but it remains elusive regarding how PAO1 responds to extracellular Pf4. Here, we report that Pf4 promotes genetic exchange among *P. aeruginosa* cells both *in vitro* and *in vivo*. Pf4 trafficking not only occurs in colonies but also can be stimulated at a much higher rate under Pf4-activating conditions. The Pf4 trafficking unveils the complex interaction between cells under the disguise of resistance and intact colonies. In addition, another filamentous prophage Pf5 also undergoes similar trafficking in *P. aeruginosa* PA14. Considering that Pf4-like filamentous phages form a large family and are widely distributed in prokaryotes and archaea [48], the observed exchange of filamentous phages might represent a common mechanism of cell–cell communications.

Biofilms display complex community interactions and functions and represent an important sessile lifestyle of almost all microorganisms [58, 59]. Although multiple forms of biofilm assays have been developed, including submerged flow cells and floating pellicles, colony growth on solid agar plates remains to be a convenient tool to study biofilm and community properties [60, 61]. The trafficking of Pf4 reveals a covert form of cooperative interaction among *P. aeruginosa* cells within the community, because the extruded extracellular Pf4 filaments confer a number of benefits to the collective *P. aeruginosa* community, including structural support for biofilms, resistance to antibiotic exposure, increased survival *in vivo* during infection by promoting biofilm formation and reducing the host inflammation and immune response [25, 26, 44, 62, 63]. An outcome of such trafficking is that the Pf4 genes are preserved within the community and may co-evolve with the host *P. aeruginosa* community. Currently, we do not find any evidence to support that Pf4 could promote the transfer of chromosomal DNA outside of the Pf4 locus but our data demonstrate the potential of Pf4-mediated genetic exchange when additional genes are inserted inside the Pf4 locus.

The production of Pf4 imposes a conceivable economic burden on producing cells [41], and existing evidence also shows that Pf4 exhibits much higher rates of mutation compared with other loci in the PAO1 genome, and chromosomal loss of Pf4 can occur due to Pf4 excision [37, 43]. This creates a conflict between the cooperative benefits and the individual burden of Pf4 within the population, which might select for Pf4 cheater cells, i.e. spontaneous Pf4-defective mutants that benefit from extracellular Pf4 protection without contribution. Similar conflicts between the group and individual benefits have been reported in various microbial models and there are multiple known strategies to reduce cheaters including kin-selection, policing, and partial privatization of public goods [64–66]. Our observation of the frequent two-way trafficking suggests an effective cheater control strategy, namely defect rescue, to prevent potential Pf4 cheating behaviors and ensure cost-sharing within the population.

**Figure 4:**
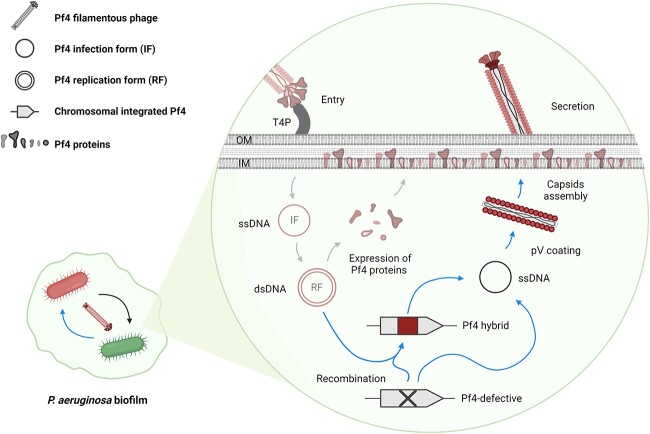
Schematic of the Pf4 two-way trafficking and defect rescue of Pf4 cheaters within *P. aeruginosa* biofilm; *P. aeruginosa* PAO1 cells continually extrude Pf4 filamentous phages in biofilm-related conditions; there is reciprocal exchange of Pf4 between *P. aeruginosa* cells in a lysis-independent and pili-dependent manner; the zoomed-in area depicts the molecular details when wild-type Pf4 is transferred into cells with a defective Pf4 and repair the Pf4 functions; the blue arrows highlight the different forms of Pf4 variants detected in this study, while the gray arrows represent mechanisms previously determined; OM, outer membrane; IM, inner membrane; T4P, type IV pili; IF, infection form; RF, replication form; pV, ssDNA binding protein PA0720.

Finally, our results suggest that the previously observed cell lysis by purified particles of Pf4 and other filamentous phages may represent an extreme event, while the reciprocal exchange of filamentous phages may better recapitulate cell–cell interactions under physiological conditions. This is especially relevant for the ∆Pf4 mutants since these are highly sensitive and prone to lysis when exposed to purified Pf4 particles [25], while our data show that the ∆Pf4 mutants can be rescued and repaired by Pf4 trafficking. In addition, the two-way trafficking of Pf4 involves sharing of both proteins and DNA between cells (Fig. 4) and enables genetic diversification through recombination and conservation. These distinct features of Pf4 exchanges share some similarities with the protein secretion systems, of which the type IV secretion system can mediate the transfer of both proteins and DNA, and the T6SS can facilitate the exchange of proteins between cells [67, 68]. Therefore, we propose that the Pf4-like trafficking resembles an additional, and yet overlooked, form of intraspecies interactions within the same community for cost sharing. Such interaction, with its capability to transfer nonphage DNA, is worth further exploring for biotechnological applications to manipulate *P. aeruginosa* in complex communities.

## Supplementary Material

Pf4_simple_wrad025

Pf4_Source_data_wrad025

## Data Availability

All data generated or analysed during this study are included in this published article and its supplementary information files.
