# Supplementary figures and images for: Filamentous prophage Pf4 promotes genetic exchange in Pseudomonas aeruginosa
Source: ISME J. 2024 Jan 10;18(1):wrad025. doi: 10.1093/ismejo/wrad025 (PMC10837833; doi:10.1093/ismejo/wrad025)

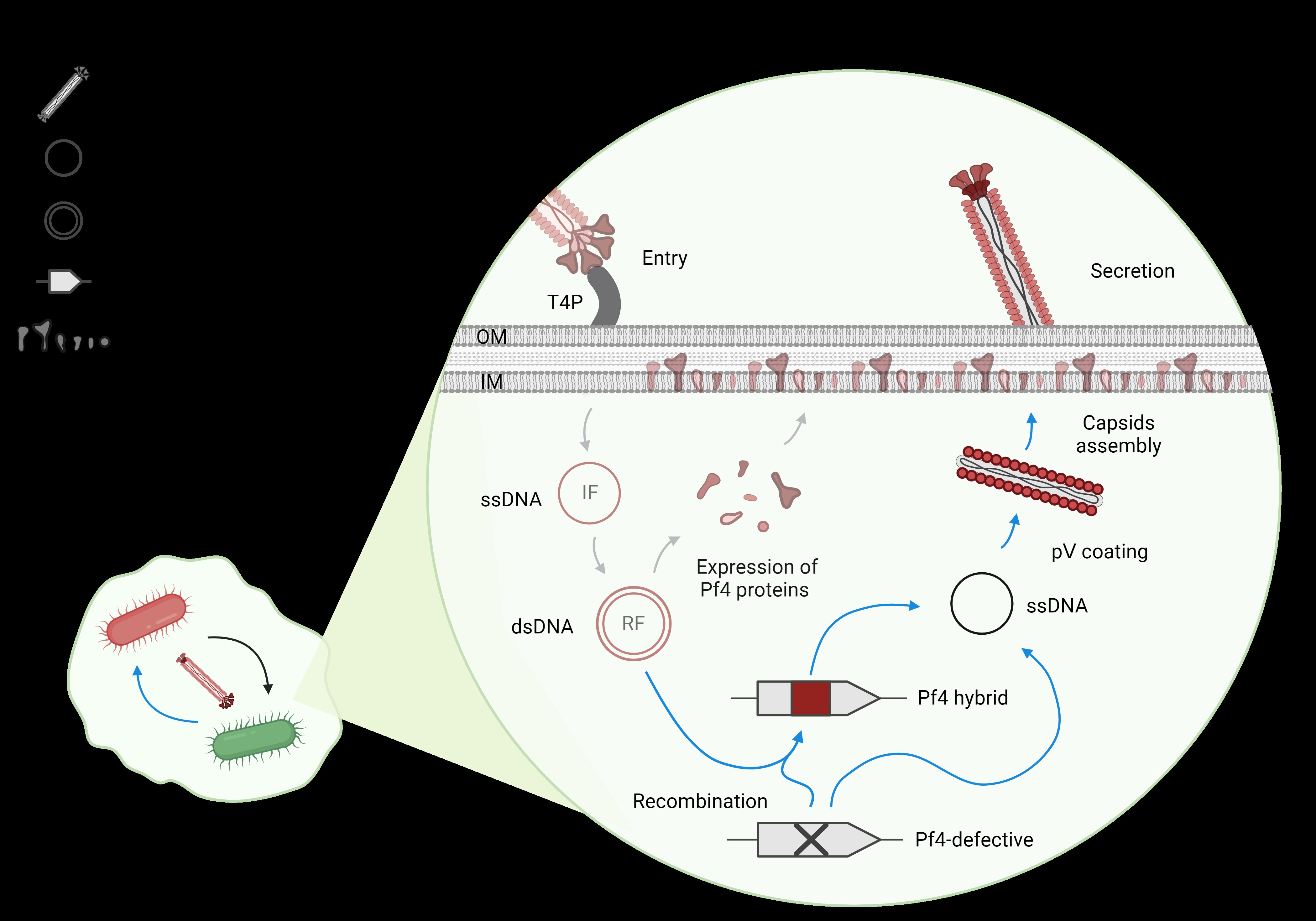

Supplement: Pf4_simple_wrad025 [file pf4_simple_wrad025.jpeg]
